# Examining the relationship between CDAI and frailty and its manifestation in Parkinson’s disease: a cross-sectional study

**DOI:** 10.3389/fnut.2024.1502748

**Published:** 2024-11-28

**Authors:** Zhaohao Zeng, Wen Jin, Kunyu Huang, Lijiao Xiong, Yu Luo, Guoyang Li, Wenli Zhang, Guo Hong, Fengju Mao, Kaifen Xiong, Xiaoguang Luo

**Affiliations:** ^1^Department of Neurology, Shenzhen People’s Hospital, The First Affiliated Hospital, Southern University of Science and Technology, The Second Clinical Medical College, Jinan University, Shenzhen, China; ^2^The Guangdong Provincial Clinical Research Center for Geriatrics, Shenzhen Clinical Research Center for Geriatrics, Shenzhen People’s Hospital, The Second Clinical Medical College, Jinan University, Shenzhen, China; ^3^Post-doctoral Scientific Research Station of Basic Medicine, Jinan University, Guangzhou, China; ^4^Department of Pharmacy, Shenshan Medical Center, Memorial Hospital of SUN YAT-SEN University, Shanwei, China; ^5^Department of Geriatrics, Shenzhen People’s Hospital, The Second Clinical Medical College, Jinan University, The First Affiliated Hospital, Southern University of Science and Technology, Shenzhen, China

**Keywords:** composite dietary antioxidant index, frailty, Parkinson’s disease, restricted cubic splines, national health and nutrition examination survey

## Abstract

**Background:**

Higher intake of antioxidants is associated with reduced risk of various chronic diseases. However, the relationship between composite dietary antioxidants and frailty has not been characterized, especially in neurodegenerative conditions like Parkinson’s disease (PD) where frailty is highly prevalent. This study aimed to investigate the association between composite dietary antioxidant index (CDAI), a composite score reflecting antioxidant vitamin and mineral intakes, and frailty risk in the general United States (US) population and PD patients.

**Methods:**

Data from 21,354 participants ≥40 years in the National Health and Nutrition Examination Survey (NHANES) 2003–2018 represented the general population sample, while 268 PD patients were analyzed separately. Frailty was defined using a validated index. Weighted logistic regression and restricted cubic splines (RCS) examined overall and nonlinear CDAI-frailty associations, adjusting for sociodemographics, lifestyle factors, and comorbidities.

**Results:**

In the general population, each unit increase in CDAI was associated with a 3.7% lower likelihood of frailty after full adjustments. Vitamin A, C, E, selenium and carotenoids exhibited J-shaped relationships where frailty risk decreased below intake thresholds of 1093.04 μg, 161.53 mg, 13.66 mg, 109.99 μg, and 5057.50 μg, respectively. In contrast, the CDAI- frailty inverse association was weaker among PD patients and only vitamin C (threshold 52.45 mg) and zinc (9.35 mg) showed nonlinear links.

**Conclusion:**

Higher dietary antioxidant intake was associated with lower frailty prevalence in the general US population, with vitamins A, C, E, selenium, and carotenoids exhibiting nonlinear J-shaped relationships. In contrast, these associations were weaker and less consistent among PD patients, with only vitamins C and zinc showing nonlinear correlations. These findings highlight population-specific differences in the role of dietary antioxidants in frailty and suggest the need for personalized nutritional strategies in PD frailty management.

## 1 Introduction

Frailty is recognized as a medical condition, with its initial definition attributed to Fried et al. (1), characterizing it as “a heightened vulnerability to homeostatic disruption following a stressful event.” As the global population ages, the prevalence of frailty is on the rise, positioning it as a growing health concern worldwide. Beyond impacting the quality of life, frailty is linked to higher mortality, increased hospital admissions, a heightened risk of falls, and the demand for prolonged care (2). This makes its prevention and management crucial for public health.

Fatigue, defined as a symptom associated with the weakening or depletion of a person’s physical and/or mental resources, constitutes a fundamental component of frailty (3–5). Often overlooked, fatigue is a non-motor symptom of Parkinson’s disease (PD). While James Parkinson identified fatigue in patients with the disease as far back as 1817, it was only in 1993 that researchers pinpointed fatigue as a concurrent condition in PD, marking it as a distinct non-motor symptom (6, 7). This symptom is among the most debilitating for those with PD (8). Remarkably, fatigue is prevalent in over half of PD (9), with studies indicating its occurrence in 33 to 70% of cases (9). This fatigue can appear early in Parkinson’s progression, even before the onset of motor symptoms, significantly affecting patients’ quality of life (10). Furthermore, while cross-sectional studies have established the high prevalence of frailty in PD patients, recent longitudinal investigations have uncovered a significant association between frailty and incident PD. This relationship persists independently of sociodemographic characteristics, lifestyle factors, multiple comorbidities, and genetic predisposition (11). The widespread nature of fatigue/frailty in Parkinson’s underscores the imperative for comprehensive research.

With the rise in global life expectancy, aging-related chronic diseases are also on the increase (12). Two predominant topics in aging research are frailty and oxidative stress (13). The latter emerges from the overproduction of reactive oxygen species (ROS) and an imbalance within the antioxidant system (12). This skewness can result in cellular damage, hastening the aging process and leading to cell death (12). Recent research indicates a strong link between frailty and oxidative stress (13, 14). Such stress can particularly impair muscle cells, causing diminished muscle function - a hallmark of frailty (15, 16). Moreover, oxidative stress ties in with inflammation, further influencing the development of frailty (17). A deeper grasp of these interactions could pave the way for novel strategies to address frailty, improving elderly health and life quality. As a result, diets high in antioxidants show promise in countering frailty and safeguarding the nervous system. The Composite Dietary Antioxidant Index (CDAI) provides a comprehensive evaluation of an individual’s intake of dietary antioxidants. It encompasses a mix of dietary antioxidants, including vitamins A, C, E, selenium, zinc, and carotenoids (18), and was crafted due to its cumulative anti-inflammatory actions against markers like TNF-*α* and IL-1β. The CDAI is not only tied to several health outcomes such as depression, overall mortality, and colorectal cancer but also acts as a vital metric for analyzing the link between antioxidant dietary consumption and overall health (15). Yet, research has not yet delved into how dietary antioxidant intake affects frailty, especially among PD sufferers.

Using a nationally representative NHANES cohort, we investigated the correlations of the dietary antioxidant index (CDAI) and its components with frailty status in the general population and PD patients. We hypothesized that higher antioxidant intakes would be associated with lower frailty risk. Additionally, we employed restricted cubic splines (RCS) to flexibly model potential nonlinear dose–response patterns, informing individualized intake recommendations. Findings from this study will help elucidate the role of dietary antioxidants in frailty etiology and provide evidence to guide nutritional guidance for managing frailty.

## 2 Materials and methods

### 2.1 Study population

**Figure 1:**
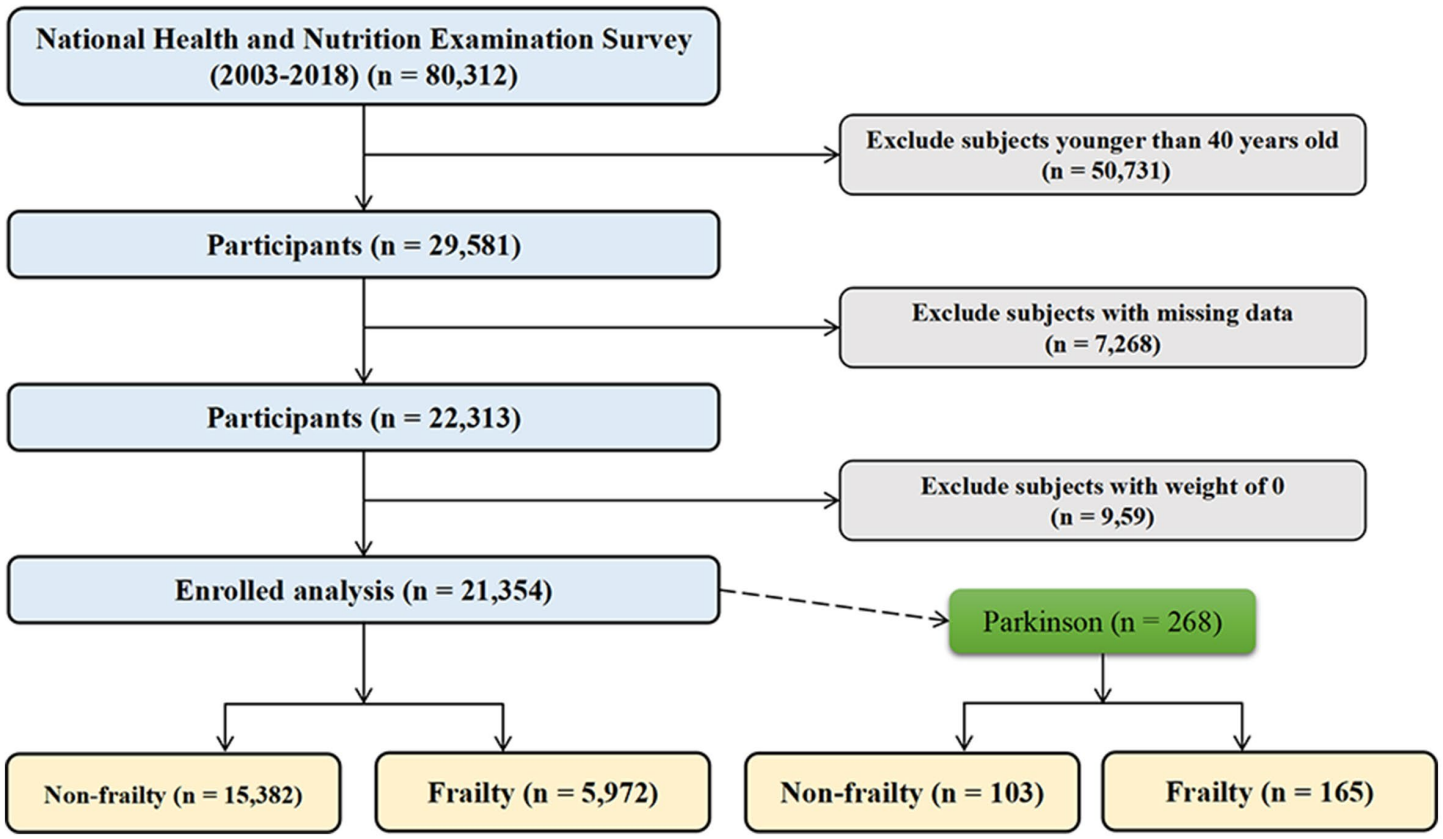
Flowchart of study population selection from the NHANES 2003–2018. This flowchart depicts the selection process for the final study population from 80,312 participants in the National Health and Nutrition Examination Survey (NHANES) cycles between 2003 and 2018.

This study utilized data from the National Health and Nutrition Examination Survey (NHANES) 2003–2018. NHANES is an ongoing cross-sectional survey conducted by the National Center for Health Statistics to assess the health and nutritional status of the US population. The surveys collect health-related questionnaires, physical examinations, and laboratory tests from a nationally representative sample. Our analysis included adults aged 40 years and older from six 2-year survey cycles (2003–2004, 2005–2006, 2007–2008, 2009–2010, 2011–2012, 2013–2014). Participants with missing data on frailty score or dietary intake were excluded. The final analytic sample comprised 21,354 participants from the general US population. In addition, 268 participants with PD were examined separately. The selection process is depicted in Figure 1.

### 2.2 Assessment of frailty

Frailty status was defined using a frailty index (FI) based on the deficit accumulation approach. The FI was computed using 49 accessible items covering symptoms, functional impairments, and comorbidities (19). Each deficit was coded as 0 or 1, with 1 indicating the presence of that deficit. The FI score was calculated by summing the number of deficits present and dividing by the total number of items, yielding a value between 0 and 1 for each participant. These multidimensional criteria evaluate frailty through an extensive array of indicators, ranging from cognitive and psychological assessments to physical performance measures, chronic disease burden, overall health evaluation, patterns of healthcare utilization, and laboratory parameters. A comprehensive breakdown of these assessment criteria is detailed in Supplementary Table S1. An FI score greater than 0.25 was used to classify individuals as frailty, while those with a score of 0.25 or below were classified as non-frailty (20–23).

### 2.3 Dietary assessment

Dietary intake reports of six antioxidants (zinc, selenium, carotenoids and vitamins A, C and E) were collected by 24-h recall, referring to 1 day prior to baseline examination. To evaluate the synergistic effects of dietary antioxidants, we used a modified version of the CDAI developed by Wright et al. (18). The CDAI was computed based on the dietary intake of zinc, selenium, carotenoids, vitamin A, vitamin C and vitamin E derived from the 24-h recall. Briefly, each of the six antioxidants was standardized by subtracting the sex-specific mean and dividing by the sex-specific standard deviation. The CDAI was then calculated by summing the standardized antioxidant intakes (24), and participants were classified into CDAI tertiles.

### 2.4 Covariate assessment

Sociodemographic covariates included age, gender, race/ethnicity, marital status, and education level. Age was categorized into 40–60 years and ≥ 60 years. Race/ethnicity was classified as non-Hispanic white, non-Hispanic black, Mexican American, and other. Marital status was defined as married/living with partner, widowed/divorced/separated, and never married. Education level was grouped into high school, below high school, and above high school. Lifestyle factors were alcohol drinking (yes/no) and smoking status (current smoker yes/no). Comorbidities included hypertension, stroke, diabetes, and PD (based on medication use) (25–29). Hypertension was defined as current antihypertensive medication use, self-reported hypertension diagnosis, or uncontrolled blood pressure > 140/90 mmHg at examination. Diabetes was determined by current insulin or diabetes medication use or HbA1c ≥6.5%. Other variables including BMI, calculated from measured weight and height, were obtained via direct measurement during the NHANES examinations, while additional factors were acquired through self-report during the interviews.

### 2.5 Statistical analysis

Baseline characteristics of the study population were stratified by frailty status (frailty vs. non- frailty) and summarized using descriptive statistics. Categorical variables were assessed using chi-square tests, while continuous variables were examined using t-tests. Categorical variables were presented as frequencies and percentages. Continuous variables were reported as means and standard deviations (SD). To explore the connection between CDAI, its constituents, and frailty, survey logistic regression models were employed. These models took into account the intricate survey design and incorporated sample weights to ensure accuracy. For the purpose of modeling potential nonlinear associations, RCS featuring four knots were utilized. These splines facilitated a flexible representation of any nonlinear relationships that might exist. A series of models were created, progressively adjusting for confounding factors. To more clearly illustrate the nonlinear relationships between CDAI, its components, and frailty risk in the RCS plots, we selected the values of CDAI and its components at the points where the curves showed the lowest odds ratios (OR) or exhibited clear inflection points as the reference points. This allowed us to more easily interpret the specific levels of these indicators where the risk of frailty was minimized or underwent notable changes. Within specific subgroups, analyses were stratified to gain deeper insights. These subgroup analyses were conducted for age (40–60 years, ≥60 years), gender (female, male), smoking status (non-smoker, smoker), and alcohol consumption (non-drinker, drinker). Analyses were adjusted for potential confounding factors, including marital status, education level, race, BMI, and chronic conditions such as hypertension and stroke. Statistical significance was established at a two-tailed P -value threshold of less than 0.05. The entirety of these analyses was executed using R version 4.2.2.

## 3 Results

### 3.1 Baseline characteristics of the study population aged 40 years and older categorized by frailty status

As shown in Table 1, a total of 21,354 participants were included, comprising 15,382 non-frailty and 5,972 frailty individuals. The mean age was 57.8 years overall, 56.7 years in the non-frailty group, and 61.5 years in the frailty group (*p* < 0.0001). Approximately half of the participants were female (51.7% overall), with a significantly higher proportion in the frailty group (60.3%) compared to the non-frailty group (51.2%) (*p* < 0.0001). The racial distribution differed between the groups (*p* < 0.0001), with non-Hispanic whites comprising 75.3% of the non-frailty and 69.8% of the frailty. A greater proportion of frailty participants were married/living with a partner (71.6% vs. 57.2%), had only a high school education (43.0% vs. 31.2%), consumed alcohol (89.2% vs. 87.4%), smoked (60.2% vs. 45.3%), and had comorbidities like hypertension, stroke, diabetes, and PD (all *p* < 0.0001). The non-frailty group had significantly higher mean BMI, cognitive disease activity index, and levels of vitamins A, C, E, zinc, selenium, and carotenoids compared to the frailty group (all *p* < 0.0001). It is worth noting that the prevalence of frailty differed significantly between the general US population and PD patients (27.97% vs. 61.57%, *p* < 0.0001).

**Table 1:** Baseline characteristics of the study population aged 40 years and older.

Variable	Total	Non-frailty	Frailty	*p*- value
Age, years	57.805 (0.166)	56.708 (0.174)	61.497(0.237)	**<0.0001**
Gender, %				**<0.0001**
Female	11,042 (51.709)	7,594 (51.225)	3,448(60.339)	
Male	10,312 (48.291)	7,788 (48.775)	2,524(39.661)	
Race, %				**<0.0001**
Non-Hispanic White	10,392 (48.665)	7,496 (75.300)	2,896(69.792)	
Non-Hispanic Black	4,520 (21.167)	3,055 (8.965)	1,465(14.174)	
Mexican American	3,042 (14.246)	2,252 (5.844)	790(6.062)	
Other Race	3,400 (15.922)	2,579 (9.891)	821(9.972)	
Marital, %				**<0.0001**
Married/Living with partner	13,464 (63.051)	10,350 (71.646)	3,114(57.168)	
Widowed/Divorced/Separated	6,261 (29.32)	3,884 (21.659)	2,377(35.714)	
Never married	1,629 (7.629)	1,148 (6.695)	481(7.118)	
Education, %				**<0.0001**
High school	7,908 (37.033)	5,334 (31.151)	2,574(43.040)	
Below high school	2,560 (11.988)	1,573 (4.372)	987(9.849)	
Over high school	10,886 (50.979)	8,475 (64.476)	2,411(47.111)	
Alcohol consumption, %				**0.015**
No	3,113 (14.578)	2,162 (10.839)	951(12.570)	
Yes	18,241 (85.422)	13,220 (89.161)	5,021(87.430)	
Smoke, %				**<0.0001**
No	10,830 (50.716)	8,284 (54.706)	2,546(39.757)	
Yes	10,524 (49.284)	7,098 (45.294)	3,426(60.243)	
BMI, kg/m^2^	29.404 (0.089)	28.757 (0.096)	31.586(0.159)	**<0.0001**
Hypertension, %				**<0.0001**
No	9,242 (43.28)	7,901 (56.023)	1,341(24.002)	
Yes	12,112 (56.72)	7,481 (43.977)	4,631(75.998)	
Stroke, %				**<0.0001**
No	20,173 (94.469)	15,044 (98.223)	5,129(87.468)	
Yes	1,181 (5.531)	338 (1.777)	843(12.532)	
Diabetes, %				**<0.0001**
No	16,269 (76.187)	12,906 (87.955)	3,363(60.726)	
Yes	5,085 (23.813)	2,476 (12.045)	2,609(39.274)	
Parkinson, %				**<0.0001**
No	21,086 (98.745)	15,279 (99.330)	5,807(96.961)	
Yes	268 (1.255)	103 (0.670)	165 (3.039)	
Population				**<0.0001**
General population	21,354	15,382	5,972	
PD patients	268	103	165	
CDAI	0.683 (0.058)	0.890 (0.063)	−0.014(0.074)	**<0.0001**
Vitamin A, μg	650.186 (9.522)	666.290 (11.285)	595.928(14.699)	**<0.001**
Vitamin C, mg	81.779 (1.187)	84.091 (1.345)	73.988(1.564)	**<0.0001**
Vitamin E, mg	8.322 (0.094)	8.626 (0.105)	7.298(0.118)	**<0.0001**
Zinc, mg	11.337 (0.101)	11.633 (0.110)	10.338(0.128)	**<0.0001**
Selenium, μg	108.942 (0.730)	111.859 (0.831)	99.111(1.190)	**<0.0001**
Carotenoid, μg	9799.624 (179.437)	10293.240 (201.230)	8136.597(237.104)	**<0.0001**

Data are presented as N% (*χ*^2^-test) or Mean (SD) (independent *t*-test). CDAI, the composite dietary antioxidant index; BMI, Body mass index. Statistically significant results are denoted by bold typeface.

### 3.2 Baseline characteristics of PD patients aged 40 years and older categorized by frailty status

The analysis included 268 PD patients, comprising 103 non-frailty and 165 frailty individuals. As shown in Table 2, the mean age was 61 years overall and did not significantly differ between the non-frailty (60.4 years) and frailty groups (61.4 years) (*p* = 0.573). There was a similar distribution of males and females in both groups (*p* = 0.59). The racial distribution was comparable between the two groups (*p* = 0.566), with the majority being non-Hispanic white (67.5% overall). Marital status, education level, and alcohol consumption were also similar between the groups (*p* > 0.05). However, a significantly higher proportion of frailty patients were smokers (57.2% vs. 37.9%, *p* = 0.04). The prevalence of comorbidities like hypertension, stroke, and diabetes was significantly higher in the frailty group compared to the non-frailty group (all *p* < 0.001). Mean BMI and levels of dietary antioxidants did not significantly differ between the two groups, except for vitamin E which was lower in the frailty group (*p* = 0.018). The cognitive disease activity index was significantly lower in the frailty (−0.627) versus the non-frailty group (0.622) (*p* = 0.006).

**Table 2:** Baseline characteristics of PD patients aged 40 years and older categorized by frailty status.

Variable	Total	Non-frailty	Frailty	*p*- value
Age, years	60.972 (1.102)	60.364 (1.636)	61.425 (1.268)	0.573
Gender, %				0.59
Female	149 (55.597)	58 (65.998)	91 (61.598)	
Male	119 (44.403)	45 (34.002)	74 (38.402)	
Race, %				0.566
Non-Hispanic White	181 (67.537)	66 (78.309)	115 (84.664)	
Non-Hispanic Black	38 (14.179)	16 (11.250)	22 (6.830)	
Mexican American	23 (8.582)	9 (4.164)	14 (3.579)	
Other Race	26 (9.701)	12 (6.277)	14 (4.926)	
Marital, %				0.293
Married/Living with partner	156 (58.209)	57 (59.891)	99 (63.381)	
Widowed/Divorced/Separated	83 (30.97)	28 (26.408)	55 (30.535)	
Never married	29 (10.821)	18 (13.701)	11 (6.083)	
Education, %				0.248
High school	105 (39.179)	40 (39.053)	65 (39.831)	
Below high school	32 (11.94)	8 (4.116)	24 (9.930)	
Over high school	131 (48.881)	55 (56.830)	76 (50.239)	
Alcohol consumption, %				0.431
No	41 (15.299)	15 (15.893)	26 (11.152)	
Yes	227 (84.701)	88 (84.107)	139 (88.848)	
Smoke, %				**0.04**
No	135 (50.373)	57 (62.060)	78 (42.760)	
Yes	133 (49.627)	46 (37.940)	87 (57.240)	
BMI, kg/m^2^	30.180 (0.581)	29.006 (1.249)	31.053 (0.848)	0.245
Hypertension, %				**<0.001**
No	88 (32.836)	55 (53.353)	33 (19.996)	
Yes	180 (67.164)	48 (46.647)	132 (80.004)	
Stroke, %				**<0.0001**
No	231 (86.194)	100 (98.041)	131 (76.669)	
Yes	37 (13.806)	3 (1.959)	34 (23.331)	
Diabetes, %				**<0.001**
No	185 (69.03)	88 (92.102)	97 (67.873)	
Yes	83 (30.97)	15 (7.898)	68 (32.127)	
CDAI	−0.094 (0.406)	0.622 (0.420)	−0.627 (0.468)	**0.006**
Vitamin A, μg	608.956 (42.629)	628.658 (64.753)	594.319 (41.541)	0.591
Vitamin C, mg	84.058 (9.608)	90.632 (7.896)	79.175 (14.797)	0.473
Vitamin E, mg	6.513 (0.419)	7.524 (0.680)	5.762 (0.389)	**0.018**
Zinc, mg	10.432 (0.511)	10.743 (0.580)	10.202 (0.693)	0.51
Selenium, μg	97.085 (5.029)	103.215 (5.275)	92.531 (6.603)	0.137
Carotenoid, μg	7474.084 (1044.686)	9054.514 (1129.760)	6299.906 (1555.171)	0.151

Data are presented as N% (*χ*^2^-test) or Mean (SD) (independent *t*-test). CDAI, the composite dietary antioxidant index; BMI, Body mass index. Statistically significant results are denoted by bold typeface.

### 3.3 Association between CDAI and frailty in adults aged over 40 years

Table 3 presents results from weighted multivariate logistic regression models examining the association between CDAI and its component dietary antioxidants with frailty in 21,354 adults aged ≥40 years. In the crude model without adjustments, higher total CDAI was associated with lower odds of frailty (OR per 1-unit increase = 0.937, 95% CI 0.924–0.949, *p* < 0.0001). This association persisted after adjusting for confounders in Models 1–3, including sociodemographics, lifestyle factors, and comorbidities (*p* for trend <0.0001 in all models). Participants in the highest quantile (Q3) of CDAI had 46% lower odds of frailty versus the lowest quantile (Q1) after full adjustments (OR = 0.684, 95% CI 0.610–0.768, *p* < 0.0001). Similar inverse dose–response relationships were observed for individual dietary antioxidants, including vitamins A, C, E, zinc, selenium, and carotenoids (all *p* for trend <0.05). In summary, higher CDAI and its component dietary antioxidants were independently associated with lower likelihood of frailty in United States adults aged ≥40 years in logistic regression models adjusting for potential demographic, lifestyle, and health confounders.

**Table 3:** Association between dietary antioxidant index (CDAI) and frailty in adults aged ≥40 years.

	Crude model		Model 1		Model 2		Model 3	
	OR (95% CI)	*p*- value	OR (95% CI)	*p*- value	OR (95% CI)	*p*- value	OR (95% CI)	*p*- value
CDAI								
Total	0.937 (0.924,0.949)	<0.0001	0.947 (0.935,0.959)	<0.0001	0.960 (0.947,0.972)	<0.0001	0.963 (0.950,0.976)	<0.0001
Q1	Ref		Ref		Ref		Ref	
Q2	0.619 (0.559,0.686)	<0.0001	0.650 (0.588,0.718)	<0.0001	0.696 (0.623,0.778)	<0.0001	0.699 (0.621,0.787)	<0.0001
Q3	0.544 (0.488,0.606)	<0.0001	0.594 (0.536,0.660)	<0.0001	0.665 (0.594,0.743)	<0.0001	0.684 (0.610,0.768)	<0.0001
*p* for trend		<0.0001		<0.0001		<0.0001		<0.0001
Vitamin A, μg								
Total (/1,000)	0.810 (0.701,0.935)	0.004	0.827 (0.718,0.954)	0.009	0.902 (0.799,1.019)	0.098	0.926 (0.824,1.041)	0.195
Q1	Ref		Ref		Ref		Ref	
Q2	0.815 (0.732,0.907)	<0.001	0.795 (0.709,0.890)	<0.001	0.837 (0.747,0.938)	0.002	0.848 (0.755,0.952)	0.006
Q3	0.699 (0.624,0.784)	<0.0001	0.711 (0.632,0.799)	<0.0001	0.802 (0.713,0.903)	<0.001	0.826 (0.733,0.931)	0.002
*p* for trend		<0.0001		<0.0001		<0.001		0.002
Vitamin C, mg								
Total (/1,000)	0.225 (0.125,0.403)	<0.0001	0.186 (0.100,0.346)	<0.0001	0.414 (0.224,0.767)	0.005	0.417 (0.225,0.771)	0.006
Q1	Ref		Ref		Ref		Ref	
Q2	0.786 (0.697,0.886)	<0.001	0.729 (0.646,0.824)	<0.0001	0.817 (0.717,0.931)	0.003	0.832 (0.727,0.953)	0.008
Q3	0.653 (0.583,0.732)	<0.0001	0.602 (0.538,0.675)	<0.0001	0.726 (0.642,0.820)	<0.0001	0.744 (0.655,0.844)	<0.0001
*p* for trend		<0.0001		<0.0001		<0.0001		<0.0001
Vitamin E, mg								
Total	0.958 (0.948,0.968)	<0.0001	0.968 (0.958,0.978)	<0.0001	0.978 (0.968,0.987)	<0.0001	0.980 (0.971,0.990)	<0.001
Q1	Ref		Ref		Ref		Ref	
Q2	0.655 (0.586,0.732)	<0.0001	0.697 (0.618,0.785)	<0.0001	0.749 (0.662,0.848)	<0.0001	0.763 (0.674,0.864)	<0.0001
Q3	0.503 (0.454,0.559)	<0.0001	0.575 (0.518,0.638)	<0.0001	0.647 (0.582,0.719)	<0.0001	0.666 (0.595,0.746)	<0.0001
*p* for trend		<0.0001		<0.0001		<0.0001		<0.0001
Zinc, mg								
Total	0.970 (0.962,0.978)	<0.0001	0.985 (0.978,0.993)	<0.001	0.986 (0.978,0.994)	<0.001	0.988 (0.980,0.995)	0.002
Q1	Ref		Ref		Ref		Ref	
Q2	0.712 (0.640,0.791)	<0.0001	0.785 (0.703,0.876)	<0.0001	0.823 (0.731,0.925)	0.001	0.833 (0.736,0.943)	0.004
Q3	0.609 (0.549,0.676)	<0.0001	0.769 (0.690,0.858)	<0.0001	0.783 (0.694,0.882)	<0.0001	0.801 (0.705,0.911)	<0.001
*p* for trend		<0.0001		<0.0001		<0.0001		<0.001
Selenium, μg								
Total	0.996 (0.995,0.997)	<0.0001	0.998 (0.997,0.999)	<0.0001	0.998 (0.996,0.999)	<0.0001	0.998 (0.996,0.999)	<0.0001
Q1	Ref		Ref		Ref		Ref	
Q2	0.719 (0.642,0.806)	<0.0001	0.797 (0.707,0.897)	<0.001	0.783 (0.688,0.890)	<0.001	0.775 (0.679,0.884)	<0.001
Q3	0.555 (0.492,0.626)	<0.0001	0.706 (0.620,0.803)	<0.0001	0.681 (0.594,0.782)	<0.0001	0.673 (0.581,0.780)	<0.0001
*p* for trend		<0.0001		<0.0001		<0.0001		<0.0001
Carotenoid, μg								
Total (/1,000)	0.983 (0.978,0.988)	<0.0001	0.985 (0.980,0.990)	<0.0001	0.989 (0.984,0.994)	<0.0001	0.989 (0.984,0.993)	<0.0001
Q1	Ref		Ref		Ref		Ref	
Q2	0.666 (0.590,0.752)	<0.0001	0.678 (0.600,0.766)	<0.0001	0.725 (0.643,0.819)	<0.0001	0.734 (0.649,0.831)	<0.0001
Q3	0.561 (0.495,0.636)	<0.0001	0.588 (0.517,0.669)	<0.0001	0.651 (0.569,0.745)	<0.0001	0.657 (0.568,0.760)	<0.0001
*p* for trend		<0.0001		<0.0001		<0.0001		<0.0001

Crudel model: Nothing confounding factors. Model 1: Adjusted for confounding factors by Age, Gender, and Race. Model 2: Adjusted for confounding factors by Age, Gender, BMI, Race, Marital, Education, Alcohol consumption, Smoke. Model 3: Adjusted for confounding factors by Age, Gender, BMI, Race, Marital, Education, Alcohol consumption, Smoke, Hypertension, Stroke. CDAI, the composite dietary antioxidant index. Specifically, the ranges for the three quartiles (Q1, Q2, Q3) of the CDAI are: Q1 [> = −7.447 and < = − 1.552], Q2 [> − 1.552 and < = 1.355], and Q3 [> 1.355 and < = 81.229]. Similarly, for the other dietary antioxidant components, the ranges are: Vitamin A - Q1 [> = 0 and < = 323] μg, Q2 [> 323 and < = 649] μg, Q3 [> 649 and < = 29,059] μg; Vitamin C - Q1 [> = 0 and < = 29.7] mg, Q2 [> 29.7 and < = 90.8] mg, Q3 [> 90.8 and < = 2,261] mg; Vitamin E - Q1 [> = 0 and < = 4.89] mg, Q2 [> 4.89 and < = 8.52] mg, Q3 [> 8.52 and < = 158.39] mg; Zinc - Q1 [> = 0 and < = 7.63] mg, Q2 [> 7.63 and < = 12.3] mg, Q3 [> 12.3 and < = 477.52] mg; Selenium - Q1 [> = 0 and < = 79] μg, Q2 [> 79 and < = 123.5] μg, Q3 [> 123.5 and < = 1195.6] μg; Carotenoids - Q1 [> = 0 and < = 2,777] μg, Q2 [> 2,777 and < = 9,045] μg, Q3 [> 9,045 and < = 377,178] μg.

### 3.4 Association between CDAI and frailty in PD aged over 40 years

Table 4 displays findings derived from weighted multivariate logistic regression models investigating the correlation between CDAI and its constituent dietary antioxidants with frailty in a cohort of 268 PD patients aged 40 years or older. In the initial model, there was a marginally significant connection, wherein higher total CDAI displayed a tendency toward reduced odds of frailty (with an odds ratio (OR) of 0.897 per 1-unit increase, accompanied by a 95% confidence interval (CI) ranging from 0.802 to 1.004, and a *p*-value of 0.058). Following the incorporation of confounding factors, this correlation became statistically significant (for Model 1, OR = 0.889, *p* = 0.039; for Model 2, OR = 0.885, *p* = 0.021). In the fully adjusted model, the topmost CDAI quantile (Q3) exhibited an 85% reduction in the odds of frailty compared to the lowest quantile (Q1) (OR = 0.142, 95% CI 0.047–0.429, *p* = 0.001). A parallel inverse dose–response pattern was identified for vitamins C and E, selenium, and carotenoids (all showing *p*-values for trend ≤0.01 in fully adjusted models). Conversely, no substantial associations were observed for vitamins A and zinc.

**Table 4:** Association between dietary antioxidant index (CDAI) and frailty in PD aged ≥40 years.

	Crude model		Model 1		Model 2		Model 3	
	OR (95% CI)	*p*- value	OR (95% CI)	*p*- value	OR (95% CI)	*p*- value	OR (95% CI)	*p*- value
CDAI								
Total	0.897 (0.802,1.004)	0.058	0.889 (0.795,0.994)	0.039	0.885 (0.799,0.981)	0.021	0.800 (0.699, 0.916)	0.002
Q1	Ref		Ref		Ref		Ref	
Q2	0.435 (0.200,0.947)	0.037	0.364 (0.162,0.815)	0.015	0.281 (0.116, 0.680)	0.006	0.313 (0.111, 0.885)	0.030
Q3	0.273 (0.121,0.616)	0.002	0.246 (0.107,0.566)	0.001	0.221 (0.095, 0.517)	<0.001	0.142 (0.047, 0.429)	0.001
*p* for trend		0.002		0.001		<0.001		<0.001
Vitamin A, μg								
Total (/1,000)	0.819 (0.395,1.701)	0.586	0.739 (0.343,1.593)	0.432	0.780 (0.378,1.610)	0.492	0.551 (0.211, 1.437)	0.215
Q1	Ref		Ref		Ref		Ref	
Q2	0.722 (0.315,1.655)	0.433	0.621 (0.268,1.441)	0.260	0.495 (0.199,1.231)	0.126	0.366 (0.115, 1.163)	0.086
Q3	0.785 (0.311,1.978)	0.600	0.659 (0.244,1.783)	0.403	0.632 (0.244,1.642)	0.336	0.419 (0.134, 1.315)	0.131
*p* for trend		0.651		0.469		0.439		0.155
Vitamin C, mg								
Total (/1,000)	0.276 (0.003,22.292)	0.558	0.164 (0.002,15.547)	0.428	0.094 (0.001,8.825)	0.298	0.002 (0.000, 0.172)	0.007
Q1	Ref		Ref		Ref		Ref	
Q2	0.327 (0.127,0.842)	0.022	0.308 (0.117,0.811)	0.018	0.235 (0.081, 0.684)	0.009	0.183 (0.062, 0.538)	0.003
Q3	0.276 (0.115,0.662)	0.005	0.237 (0.096,0.585)	0.002	0.190 (0.073, 0.495)	0.001	0.170 (0.055, 0.525)	0.003
*p* for trend		0.005		0.002		0.001		0.004
Vitamin E, mg								
Total	0.894 (0.823,0.971)	0.009	0.883 (0.812,0.960)	0.005	0.878 (0.801,0.961)	0.006	0.846 (0.761, 0.942)	0.003
Q1	Ref		Ref		Ref		Ref	
Q2	0.472 (0.214,1.039)	0.062	0.425 (0.190,0.950)	0.038	0.454 (0.189,1.092)	0.076	0.379 (0.143, 1.003)	0.051
Q3	0.324 (0.131,0.797)	0.015	0.286 (0.114,0.720)	0.009	0.246 (0.088,0.692)	0.009	0.210 (0.072, 0.614)	0.006
*p* for trend		0.013		0.007		0.008		0.005
Zinc, mg								
Total	0.983 (0.931,1.038)	0.524	0.977 (0.922,1.035)	0.425	0.981 (0.921,1.045)	0.545	0.975 (0.917, 1.037)	0.413
Q1	Ref		Ref		Ref		Ref	
Q2	0.678 (0.242,1.897)	0.452	0.671 (0.233,1.928)	0.449	0.774 (0.274,2.186)	0.620	0.581 (0.209, 1.616)	0.288
Q3	0.597 (0.273,1.308)	0.192	0.559 (0.249,1.253)	0.153	0.605 (0.257,1.426)	0.243	0.498 (0.195, 1.268)	0.139
*p* for trend		0.189		0.15		0.245		0.135
Selenium, μg								
Total	0.996 (0.990,1.002)	0.159	0.995 (0.989,1.001)	0.090	0.994 (0.987,1.000)	0.055	0.990 (0.983, 0.998)	0.014
Q1	Ref		Ref		Ref		Ref	
Q2	0.321 (0.137,0.750)	0.010	0.308 (0.132,0.720)	0.008	0.273 (0.121, 0.617)	0.003	0.336 (0.136, 0.830)	0.020
Q3	0.592 (0.278,1.257)	0.168	0.554 (0.249,1.234)	0.144	0.503 (0.214, 1.180)	0.111	0.357 (0.137, 0.931)	0.036
*p* for trend		0.074		0.058		0.037		0.02
Carotenoid, μg								
Total (/1,000)	0.975 (0.921,1.031)	0.364	0.971 (0.919,1.026)	0.283	0.970 (0.926,1.017)	0.196	0.928 (0.886, 0.971)	0.002
Q1	Ref		Ref		Ref		Ref	
Q2	0.251 (0.112,0.564)	0.001	0.245 (0.107,0.562)	0.001	0.251 (0.104,0.606)	0.003	0.222 (0.081, 0.605)	0.004
Q3	0.281 (0.115,0.684)	0.006	0.256 (0.108,0.609)	0.003	0.250 (0.107,0.580)	0.002	0.192 (0.068, 0.548)	0.003
*p* for trend		0.003		<0.001		<0.001		0.002

Crudel model: Nothing confounding factors. Model 1: Adjusted for confounding factors by Age, Gender, Race. Model 2: Adjusted for confounding factors by Age, Gender, BMI, Race, Marital, Education, Alcohol consumption, Smoke. Model 3: Adjusted for confounding factors by Age, Gender, BMI, Race, Marital, Education, Alcohol consumption, Smoke, Hypertension, Stroke.CDAI, the composite dietary antioxidant index. Specifically, the ranges for the three quartiles (Q1, Q2, Q3) of the CDAI are: Q1 [> = −7.447 and < = − 1.552], Q2 [> − 1.552 and < = 1.355], and Q3 [> 1.355 and < = 81.229]. Similarly, for the other dietary antioxidant components, the ranges are: Vitamin A - Q1 [> = 0 and < = 323] μg, Q2 [> 323 and < = 649] μg, Q3 [> 649 and < = 29,059] μg; Vitamin C - Q1 [> = 0 and < = 29.7] mg, Q2 [> 29.7 and < = 90.8] mg, Q3 [> 90.8 and < = 2,261] mg; Vitamin E - Q1 [> = 0 and < = 4.89] mg, Q2 [> 4.89 and < = 8.52] mg, Q3 [> 8.52 and < = 158.39] mg; Zinc - Q1 [> = 0 and < = 7.63] mg, Q2 [> 7.63 and < = 12.3] mg, Q3 [> 12.3 and < = 477.52] mg; Selenium - Q1 [> = 0 and < = 79] μg, Q2 [> 79 and < = 123.5] μg, Q3 [> 123.5 and < = 1195.6] μg; Carotenoids - Q1 [> = 0 and < = 2,777] μg, Q2 [> 2,777 and < = 9,045] μg, Q3 [> 9,045 and < = 377,178] μg.

### 3.5 Stratified analysis of the association between CDAI and frailty by age, gender, smoking, and alcohol consumption

Table 5 displays findings sourced from segmented multivariate logistic regression models that explore the connection between CDAI and frailty within distinct subgroups. These models were adjusted for variables such as marital status, education, race, BMI, and concurrent conditions like diabetes, stroke, and hypertension. Elevated CDAI levels were linked to a reduced probability of experiencing frailty across both age categories, demonstrating a somewhat more pronounced correlation in the 40–60 years bracket (with an odds ratio (OR) of 0.946 per 1-unit increase, accompanied by a 95% confidence interval (CI) ranging from 0.928 to 0.965, and a *p*-value below 0.0001) as opposed to the ≥60 years category (OR = 0.967, 95% CI 0.950–0.985, *p* < 0.001), although the interaction lacked significance (*p* = 0.085). This inverse relationship between CDAI and frailty persisted across both genders, showcasing a trend toward a slightly more potent association in males (OR = 0.943, *p* < 0.0001) compared to females (OR = 0.967, *p* < 0.0001) (interaction p-value = 0.059). Moreover, increased CDAI values correlated with diminished odds of frailty among both non-smokers (OR = 0.966, *p* < 0.001) and smokers (OR = 0.952, *p* < 0.0001), as well as non-drinkers (OR = 0.974, *p* = 0.099) and drinkers (OR = 0.953, *p* < 0.0001), without any substantial interaction effects. In summary, heightened CDAI levels were linked to decreased odds of experiencing frailty in all the examined subgroups, with no notable variances by age, gender, smoking habits, or alcohol consumption.

**Table 5:** Stratified analysis of the association between dietary antioxidant index (CDAI) and frailty by age, gender, smoking, and alcohol consumption.

Character	OR (95% CI)	*p*- value	*p* for interaction
Age			0.085
40–60 years old	0.946 (0.928,0.965)	<0.0001	
>= 60 years old	0.967 (0.950,0.985)	<0.001	
Gender			0.059
Female	0.967 (0.951,0.982)	<0.0001	
Male	0.943 (0.922,0.966)	<0.0001	
Smoke			0.372
No	0.966 (0.949,0.984)	<0.001	
Yes	0.952 (0.932,0.972)	<0.0001	
Alcohol consumption			0.185
No	0.974 (0.945,1.005)	0.099	
Yes	0.953 (0.939,0.967)	<0.0001	

Adjusted for confounding factors by Marital, Education, Race, BMI and chronic disease including hypertension and Stroke.

### 3.6 Stratified analysis of the association between CDAI and frailty in PD patients

Table 6 presents results from stratified multivariate logistic regression models examining the association between CDAI and frailty within subgroups of 268 PD patients. In the 40–60 years age group, higher CDAI was associated with lower likelihood of frailty (OR per 1-unit increase = 0.864, 95% CI 0.749–0.996, *p* = 0.045), but this association was not significant in the ≥60 years group (OR = 0.848, *p* = 0.104), without a significant interaction (*p* = 0.925). A significant inverse association was observed between CDAI and frailty in women (OR = 0.756, *p* = 0.005) but not men (OR = 0.982, *p* = 0.797), with a significant interaction (*p* = 0.028). Higher CDAI was associated with lower odds of frailty in smokers (OR = 0.719, *p* = 0.004) but not non-smokers (OR = 0.969, *p* = 0.639), with a significant interaction (*p* = 0.018). The association was also significant in drinkers (OR = 0.857, *p* = 0.014) but not non-drinkers (OR = 0.906, *p* = 0.565), without a significant interaction (*p* = 0.189).

**Table 6:** Stratified analysis of the association between dietary antioxidant index (CDAI) and frailty in PD patients.

Character	OR (95% CI)	*p*- value	*p* for interaction
Age			0.925
40–60 years old	0.864 (0.749, 0.996)	0.045	
>= 60 years old	0.848 (0.676, 1.064)	0.104	
Gender			0.028
Female	0.756 (0.629, 0.908)	0.005	
Male	0.982 (0.838, 1.151)	0.797	
Smoke			0.018
No	0.969 (0.838, 1.121)	0.639	
Yes	0.719 (0.588, 0.879)	0.004	
Alcohol consumption			0.189
No	0.906 (0.557, 1.475)	0.565	
Yes	0.857 (0.760, 0.967)	0.014	

Adjusted for confounding factors by Marital, Education, Race, BMI and chronic disease including hypertension and Stroke.

### 3.7 Nonlinear dose–response relationships between CDAI, its components, and frailty risk

**Figure 2:**
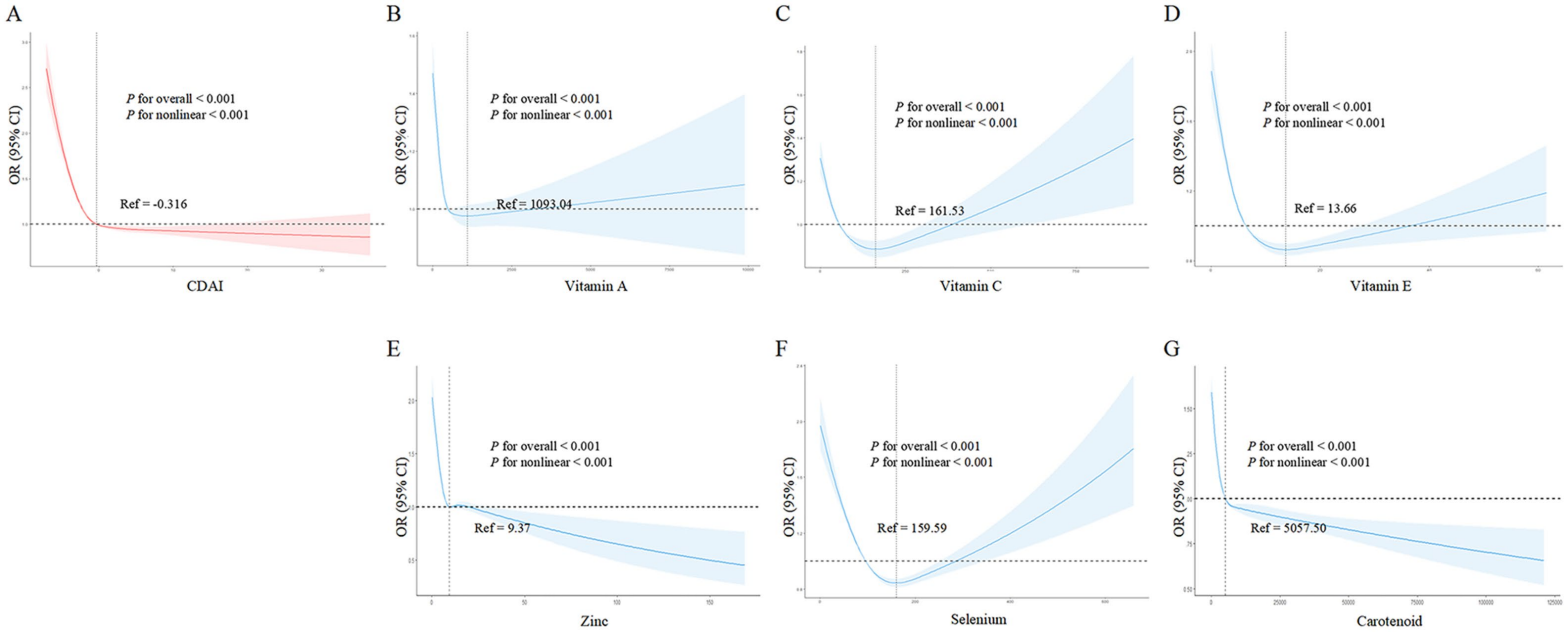
Nonlinear dose - response relationships between dietary antioxidant index (CDAI), its components, and frailty risk in the general population. **(A)** CDAI; **(B)** Vitamin A; **(C)** Vitamin C; **(D)** Vitamin E; **(E)** Zinc; **(F)** Selenium; **(G)** Carotenoids. Restricted cubic spline analysis was used to examine the nonlinear associations between CDAI, its components, and frailty risk, adjusting for age, gender, BMI, race, marital status, education, alcohol, smoking, hypertension, and stroke.

Figure 2A shows that as CDAI increased, frailty risk decreased rapidly until a CDAI of −0.316, beyond which CDAI became protective against frailty, conferring a gradual reduction in frailty risk (*p*- overall <0.001, *p*- nonlinear <0.001). Similarly, vitamins A (Figure 2B), C (Figure 2C), E (Figure 2D), and selenium (Figure 2F) exhibited J-shaped relationships with frailty risk. For vitamin A, frailty risk decreased sharply until 1093.04 μg intake, beyond which risk increased again (*p*- overall <0.001, *p*- nonlinear <0.001). For vitamin C, this threshold was 161.53 mg (*p*- overall <0.001, *p*- nonlinear <0.001). For vitamin E, risk decreased until 13.66 mg intake, and for selenium until 109.99 μg (both *p*- overall <0.001, *p*- nonlinear <0.001). In contrast, zinc (Figure 2E) and carotenoids (2G) showed inverse linear associations, conferring frailty risk reduction starting from thresholds of 9.37 mg and 5057.50 μg, respectively (both *p*- overall <0.001, *p*- nonlinear <0.001). In summary, nonlinear U-or J-shaped relationships were revealed between antioxidant vitamins/minerals and frailty risk, highlighting potential optimal intake levels.

### 3.8 Nonlinear dose–response relationships between CDAI, its components, and frailty risk in PD

**Figure 3:**
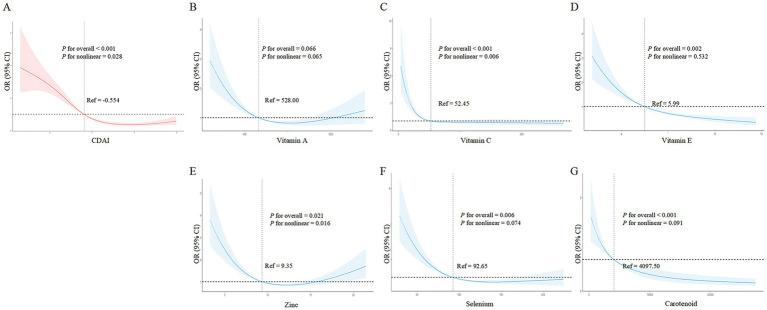
Nonlinear dose–response relationships between dietary antioxidant index (CDAI), its components, and frailty risk in PD patients. **(A)** CDAI; **(B)** Vitamin A; **(C)** Vitamin C; **(D)** Vitamin E; **(E)** Zinc; **(F)** Selenium; **(G)** Carotenoids. Restricted cubic spline analysis was used to examine the nonlinear associations between CDAI, its components, and frailty risk, adjusting for age, gender, BMI, race, marital status, education, alcohol, smoking, hypertension, and stroke.

Similar to the general population, Figure 3A shows that higher CDAI was associated with lower frailty risk, becoming protective against frailty after a CDAI of −0.554. Among CDAI components, only vitamin C intake (Figure 3C) and zinc (Figure 3E) exhibited nonlinear associations with frailty risk (vitamin C: *p*- overall <0.001, *p*- nonlinear =0.006; zinc: *p*- overall = 0.021, *p*- nonlinear =0.016). Higher intakes of vitamin C and zinc were accompanied by decreased frailty risk. Vitamin C became protective against frailty after 52.45 mg, while for zinc this threshold was 9.35 mg. Notably, frailty risk began to increase slightly again beyond 15 mg of zinc. Other components including vitamins A, E, selenium, and carotenoids (Figures 3B,D,F,G) did not show evidence of nonlinear correlations with frailty risk (all *p*- nonlinear >0.05). However, higher intakes of vitamin E, selenium, and carotenoids appeared to confer lower frailty risk (*p*- overall <0.05). In summary, nonlinear relationships were revealed for vitamin C and zinc in relation to frailty within PD patients.

## 4 Discussion

In this large, nationally representative study, we found novel evidence that higher CDAI and its component antioxidants are associated with lower likelihood of frailty in the general US population aged 40 years and older. Several antioxidants including vitamins A, C, E, selenium and carotenoids exhibited nonlinear J-shaped correlations with frailty risk, highlighting potential optimal intake levels. These findings persisted after adjusting for various sociodemographic, lifestyle, and health factors. Notably, these associations were weaker and less consistent among PD patients, suggesting potential disease-specific differences in the role of dietary antioxidants in frailty etiology and management. The antioxidant-frailty associations were notably attenuated and less consistent in this patient population, suggesting that the complex pathophysiology of PD may modulate the impact of dietary antioxidants on frailty. These differential findings underscore the need for a nuanced, disease-specific approach to understanding the role of dietary antioxidants in frailty etiology and management, particularly in the context of neurodegenerative conditions like PD.

Our analyses revealed different patterns of antioxidant-frailty associations between the general population and PD patients. In the general population, higher CDAI was consistently associated with lower frailty prevalence. All studied antioxidants (vitamins A, C, E, zinc, selenium, and carotenoids) showed protective effects against frailty. However, among PD patients, the CDAI-frailty association was notably weaker, with only vitamins C and E, selenium, and carotenoids showing protective effects. The dose–response patterns also differed between populations. In the general population, several antioxidants showed J-shaped relationships with frailty risk, suggesting optimal intake ranges. In contrast, PD patients showed simpler relationships, with only vitamin C and zinc demonstrating nonlinear associations. These distinct patterns suggest that dietary antioxidants may work differently in PD patients compared to the general population, highlighting the need for disease-specific approaches to nutritional intervention.

Previous studies have suggested a potential link between frailty and oxidative stress. Oxidative stress results from an imbalance between reactive oxygen species and antioxidants, leading to cellular damage. This imbalance may contribute to the pathophysiology underlying frailty in the elderly. Some researchers propose that oxidative stress-induced damage could be an early event in the development of frailty, as it may lead to the multiple alterations seen in frail individuals (30, 31). Furthermore, oxidative stress has been implicated as a key mechanism in neurodegenerative diseases like Alzheimer’s disease and PD (13), suggesting it may significantly impact the progression of frailty symptoms. Epidemiological studies reveal an association between physical frailty and cognitive decline, with mitochondrial dysfunction and oxidative stress as potential common denominators (32, 33). Given the role of oxidative stress in neurodegenerative diseases, antioxidant supplementation may help ameliorate cognitive impairment and thereby an important component of frailty (34). Further research is warranted to elucidate the relationship between oxidative stress, frailty, and neurodegenerative disease. Antioxidant therapy may provide a potential avenue for prevention or treatment of frailty, particularly the cognitive aspects, in aging populations.

Handgrip strength serves as a valuable diagnostic tool for assessing frailty. Prior investigations have consistently demonstrated a strong positive correlation between handgrip strength and the CDAI. Notably, the intake of antioxidant nutrients such as vitamin E, selenium, and zinc has been linked to enhanced handgrip strength among men, while for women, improved handgrip strength is specifically associated with zinc intake (15). An in-depth exploration of dietary status and frailty among elderly Koreans revealed that elevated consumption of vitamin D, vitamin C, and folate holds the potential to mitigate frailty and diminish the risk of malnutrition in the aging population (35). Similar findings have been replicated in various cross-sectional studies (36). Furthermore, a separate cross-sectional study unveiled that individuals with robust health exhibit higher levels of blood carotenoids in comparison to frail or pre-frail counterparts. This underscores the potential utility of carotenoid concentrations as a blood biomarker for assessing frailty (37). In the context of a prospective Australian study, it was established that inadequate intake of antioxidants, particularly vitamin E, significantly correlates with frailty in elderly men. The investigation also indicated that supplementation with low-dose antioxidants could be a promising strategy for preventing frailty (38). Our study undertook a comprehensive analysis of the collective impacts of these dietary antioxidants and identified a positive relationship between heightened total antioxidant intake and reduced frailty. Furthermore, we observed that insufficient intake of selenium and zinc corresponded to an increased risk of frailty. Some research has indicated that diminished serum selenium levels are linked to reduced muscle mass in elderly women (39), as well as diminished handgrip strength among older females (40). The deficiency of zinc may impair antioxidant functionality, potentially contributing to muscle loss and frailty. However, the relationship between zinc and frailty requires further substantiation through additional evidence (41). Within our study, increased intake of zinc and carotenoids was associated with reduced frailty, while other antioxidants, including vitamins A, C, and E, as well as selenium, demonstrated efficacy in alleviating frailty only when consumed below specific threshold levels.

Degeneration of dopaminergic neurons is the core pathology in Parkinson’s disease, and oxidative stress is a major contributing factor to this neurodegeneration (42). Deficiencies in some antioxidant vitamins have been shown to associate with increased PD risk, and may even contribute to PD onset and progression, such as carotenoids, vitamin A, and vitamin C (43–45). Previous studies show vitamin A intake can effectively protect dopaminergic neurons and improve motor impairment in PD rat models *in vitro* (46). However, contradictory results exist, as supplementing vitamin A may also promote oxidative stress, increase *α*-synuclein phosphorylation, elevate oxidative stress levels, and facilitate neuronal death (47). Some cohort studies have struggled to elucidate correlations between vitamin A and PD risk, indicating the role of vitamin A in neuroprotection remains unclear (48, 49). Our study found vitamin A exhibited a possible U-shaped relationship with PD frailty improvement, though not statistically significant. Vitamin E deficiency may lead to dopaminergic neuron degeneration (44), and dietary vitamin E intake associates with reduced PD risk (50). Similarly, dietary zinc intake correlates with decreased PD risk (51). Basic research has confirmed selenium as a protective factor for PD. For example, sodium selenite can dose-dependently reverse the decrease in dopamine and metabolites induced by MPTP (52). A selenium-deficient diet can enhance methamphetamine-induced tyrosine hydroxylase-like immunoreactivity in the substantia nigra, reduce dopamine and metabolites, exacerbate loss of dopaminergic neurons, while a selenium-rich diet can significantly mitigate methamphetamine neurotoxicity in dopaminergic neurons (53). Although various dietary antioxidants demonstrate neuroprotective effects in PD, no studies have examined associations between dietary antioxidants and PD frailty. Our results show increased dietary zinc and carotenoid intakes associate with reduced frailty risk in a dose-dependent manner. Additionally, total dietary antioxidant intake may correlate with improved PD frailty.

In summary, for the general population, our findings suggest that adopting a diet rich in these key antioxidant nutrients may help prevent or manage frailty. This could involve increasing consumption of fruits, vegetables, nuts, seeds, and other antioxidant-dense foods. In contrast, the associations between dietary antioxidants and frailty were weaker and less consistent among PD patients. This suggests that the management of frailty in PD may require a more nuanced, disease-specific approach that goes beyond just optimizing antioxidant intake. Personalized nutritional strategies combined with other therapeutic approaches may be most beneficial for this patient population.

This study has several notable strengths. Firstly, the large, nationally representative NHANES dataset allowed analysis of over 21,000 adults to provide robust insights into antioxidant-frailty associations in the general United States population. Furthermore, the availability of detailed dietary data enabled construction of the composite Dietary Antioxidant Index as well as investigation of individual antioxidant components. Other strengths include adjustment for a comprehensive set of confounders, measurement of frailty using a validated scale, and novel use of flexible nonlinear methods to uncover potential optimal intake levels.

Despite these strengths, our study has several limitations that warrant consideration. First, the cross-sectional design precludes causal inferences about the relationship between dietary antioxidants and frailty. Although we adjusted for numerous potential confounders, residual confounding by unmeasured factors such as physical activity, sleep quality, and medication use cannot be ruled out. Second, dietary intake was assessed using 24-h recalls, which may not capture long-term or usual intake patterns and are susceptible to recall bias. However, the use of trained interviewers and standardized protocols in NHANES helps mitigate these issues. Third, while the frailty index is a validated measure of overall health status, it may not fully capture all dimensions of frailty. Future studies using more specific frailty scales are needed to confirm our findings. Fourth, the relatively small sample size of PD patients may have limited statistical power to detect associations in this subgroup, especially for the rarer antioxidant nutrients. Finally, the lack of longitudinal follow-up data precluded assessment of temporal relationships and changes in frailty status over time. Prospective cohort studies with repeated measures of diet and frailty are needed to better understand the long-term effects of dietary antioxidants on frailty risk and progression.

## 5 Conclusion

In conclusion, this is the first study elucidating the link between dietary total antioxidant intake and frailty risk, while also revealing differences between the general and PD populations. This large nationally representative study provides novel evidence linking higher dietary antioxidant intake to lower frailty risk in the general US population aged 40 and older. We found significant inverse associations between the CDAI and several individual antioxidant nutrients, including vitamins A, C, E, selenium, and carotenoids, with frailty prevalence. Notably, these associations were weaker and less consistent among PD patients, suggesting potential disease-specific differences in the role of dietary antioxidants in frailty etiology and management.

## Data Availability

Publicly available datasets were analyzed in this study. This data can be found at: https://www.cdc.gov/nchs/nhanes/index.htm.
