# Supplementary material for: Examining the relationship between CDAI and frailty and its manifestation in Parkinson’s disease: a cross-sectional study
Source: Front Nutr. 2024 Nov 28;11:1502748. doi: 10.3389/fnut.2024.1502748 (PMC11635959; doi:10.3389/fnut.2024.1502748)
Supplement: Supplementary file 1 [file Table_1.DOCX]

**Supplementary Table 1**. Detailed Scoring Criteria and Components of the 49-Item Frailty Index

| **Defects** | **Scoring** |
| --- | --- |
| **Cognition** | |
| 1. Experience confusion/memory problems | Yes = 1  No = 0 |
| **Dependence** | |
| 2. Managing money | Difficulty = 1  No Difficulty = 0 |
| 3. Stooping, crouching, kneeling |  |
| 4. Lifting or carrying |  |
| 5. House chore |  |
| 6. Preparing meals |  |
| 7. Standing up from armless chair |  |
| 8. Getting in and out of bed difficulty |  |
| 9. Using fork, knife, drinking from cup |  |
| 10. Dressing yourself |  |
| 11. Standing for long periods difficulty |  |
| 12. Grasp/holding small objects |  |
| 13. Attending social events |  |
| 14. Push or pull large objects |  |
| 15. Walking for a quarter mile difficulty |  |
| 16. Walking up 10 steps difficulty |  |
| **Depressive Symptoms** | |
| 17. Have little interest in doing things | Nearly every day = 1  More than half the days = 0.66  Several days = 0.33  Not at all = 0 |
| 18. Feeling down, depressed, or hopeless |  |
| 19. Trouble sleeping or sleeping too much |  |
| 20. Feeling tired or having little energy |  |
| 21. Poor appetite or overeating |  |
| 22. Feeling bad about yourself |  |
| 23. Trouble concentrating on things |  |
| **Comorbidities** | |
| 24. Arthritis | Yes = 1  Suspect = 0.5  No = 0 |
| 25. Thyroid problems |  |
| 26. Chronic bronchitis |  |
| 27. Cancer |  |
| 28. Congestive heart failure |  |
| 29. Coronary heart disease |  |
| 30. Angina |  |
| 31. Heart attack |  |
| 32. Stroke |  |
| 33. Blood pressure |  |
| 34. Diabetes |  |
| 35. Weak/failing kidneys |  |
| 36. Urinary Leakage |  |
| **Hospital Utilization and Access to Care** | |
| 37. Self-rated health | Fair or poor = 1  Excellent, very good, or good = 0 |
| 38. Health now compared with 1 year ago | Worse = 1  About the same, Better = 0 |
| 39. Overnight hospital patient in past year | Yes = 1  No = 0 |
| 40. Frequency of healthcare use during the past year | None = 0, 1 to 5 = 0.5, More than 5 = 1 |
| 41. Number of prescribed medications | None = 0, 1 to 4 = 0.5, ≥5 = 1 |
| **Physical Performance and Anthropometry** | |
| 42. Body mass index | <18.5 or ≥30 = 1  25 to 30 = 0.5  18.5 to 25 = 0 |
| 43. Handgrip strength | Male:  For BMI ≤ 24, GS ≤ 29 = 1;  For BMI 24 to 28, GS ≤ 30 = 1;  For BMI >28, GS ≤ 32 = 1.  Female:  For BMI ≤23, GS ≤17 = 1;  For BMI 23 to 26, GS ≤17.3 = 1;  For BMI 26 to 29, GS ≤ 18 = 1;  For BMI>29, GS ≤ 21 = 1. |
| **Laboratory Values** | |
| 44. Glycohemoglobin (%) | 0% to 5.7% = 0, >5.7% = 1 |
| 45. Red blood cell count **(million cells/ml)** | Male: 4.7 to 6.1 = 0, Other = 1  Female: 4.2 to 5.4 = 0, Other = 1 |
| 46. Hemoglobin (g/dL) | Male: 13.5 to 18 = 0, Other = 1  Female: 12 to 16 = 0, Other = 1 |
| 47. Red cell distribution width (%) | 11.6 to 14.6 = 0, Other = 1 |
| 48. Lymphocyte percent (%) | 20 to 40 = 0, Other = 1 |
| 49. Segmented neutrophils percent (%) | 40 to 80 = 0, Other = 1 |

BMI, Body mass index; GS, grip strength.
